# Supplementary material for: A fault-intrusion-tolerant system and deadline-aware algorithm for scheduling scientific workflow in the cloud
Source: PeerJ Comput Sci. 2021 Nov 2;7:e747. doi: 10.7717/peerj-cs.747 (PMC8576554; doi:10.7717/peerj-cs.747)
Supplement: Supplemental Information 6 [file peerj-cs-07-747-s006.zip › Myproject1.1/docs/org/workflowsim/clustering/AbstractArrayList.html]

AbstractArrayList


JavaScript is disabled on your browser.


- Overview
- Package
- Class
- Use
- Tree
- Deprecated
- Index
- Help

- Prev Class
- Next Class

- Frames
- No Frames

- All Classes

- Summary:
- Nested |
- Field |
- Constr |
- Method

- Detail:
- Field |
- Constr |
- Method


org.workflowsim.clustering

## Class AbstractArrayList

- java.lang.Object
- - org.workflowsim.clustering.AbstractArrayList

- ---

    

  ```
  public class AbstractArrayList
  extends java.lang.Object
  ```

  This data structure AbstractArrayList is used in clustering alone It is
  better than ArrayList since it associates with depth information

  Since:
  :   WorkflowSim Toolkit 1.0

- - ### Field Summary

    Fields

    | Modifier and Type | Field and Description |
    | --- | --- |
    | `boolean` | `hasChecked` A check point. |
  - ### Constructor Summary

    Constructors

    Constructor and Description

    `AbstractArrayList(java.util.ArrayList taskList,
    int depth)`

    Initialize AbstractArrayList
  - ### Method Summary

    Methods

    | Modifier and Type | Method and Description |
    | --- | --- |
    | `java.util.ArrayList` | `getArrayList()` Gets the task list |
    | `int` | `getDepth()` Gets the depth of these tasks |

    - ### Methods inherited from class java.lang.Object

      `clone, equals, finalize, getClass, hashCode, notify, notifyAll, toString, wait, wait, wait`

- - ### Field Detail


    - #### hasChecked

      ```
      public boolean hasChecked
      ```

      A check point.
  - ### Constructor Detail


    - #### AbstractArrayList

      ```
      public AbstractArrayList(java.util.ArrayList taskList,
                       int depth)
      ```

      Initialize AbstractArrayList

      Parameters:
      :   `taskList` - the task list
      :   `depth` - the level of these tasks
  - ### Method Detail


    - #### getArrayList

      ```
      public java.util.ArrayList getArrayList()
      ```

      Gets the task list

      Returns:
      :   task list


    - #### getDepth

      ```
      public int getDepth()
      ```

      Gets the depth of these tasks

      Returns:
      :   depth


- Overview
- Package
- Class
- Use
- Tree
- Deprecated
- Index
- Help

- Prev Class
- Next Class

- Frames
- No Frames

- All Classes

- Summary:
- Nested |
- Field |
- Constr |
- Method

- Detail:
- Field |
- Constr |
- Method
